# Anion Exchange Membrane Water Electrolysis at 10 A ⋅ cm^−2^ Over 800 Hours

**DOI:** 10.1002/anie.202413698

**Published:** 2024-11-07

**Authors:** Yiwei Zheng, Wenchao Ma, Ariana Serban, Andrit Allushi, Xile Hu

**Affiliations:** ^1^ Laboratory of Inorganic Synthesis and Catalysis Institute of Chemical Sciences and Engineering Ecole Polytechnique Fédérale de Lausanne (EPFL) Lausanne CH-1015 Switzerland

**Keywords:** anion exchange membrane, water electrolyzer, high current density, anode, interfacial engineering

## Abstract

Anion exchange membrane water electrolyzer (AEMWE) is a potentially cost‐effective technology for green hydrogen production. Although the normal current densities of AEMWEs are below 3 A ⋅ cm^−2^, operating them at higher current densities represents an efficient, but little‐explored approach to decrease the total cost of hydrogen production. We show here that a benchmark AEMWE has an operational lifetime of only seconds at an ultrahigh current density of 10 A ⋅ cm^−2^. By using a more conductive and robust AEM, and judicious choices of ionomers, catalyst, and porous transport layer, we have developed AEMWEs that stably operate at 10 A ⋅ cm^−2^ with extended lifetimes. The optimized AEMWE has an operational lifetime of more than 800 hours, a 5‐order magnetite improvement over the current benchmark. The cell voltage is only 2.3 V at 10 A ⋅ cm^−2^, comparable to those of the state‐of‐the‐art devices operating at current densities lower than 3 A ⋅ cm^−2^. This work demonstrates the potential of ultrahigh current density AEMWEs.

## Graphical abstract

An efficient anion exchange membrane (AEM) and judicious choices of ionomers, catalyst, and porous transport layer enable an AEM water electrolysizer (AEMWE) to operate at an ultrahigh current density of 10 A ⋅ cm^−2^ at 2.3 V. The optimized AEMWE demonstrates an operational lifetime of more than 800 hours, a 5‐order magnetite improvement over a benchmark.

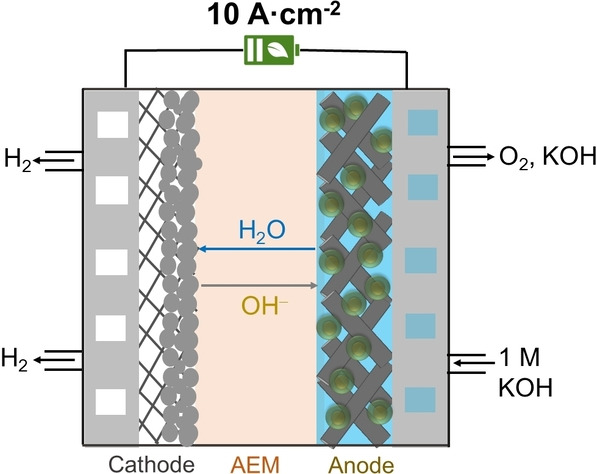

## Introduction

**Figure 1:**
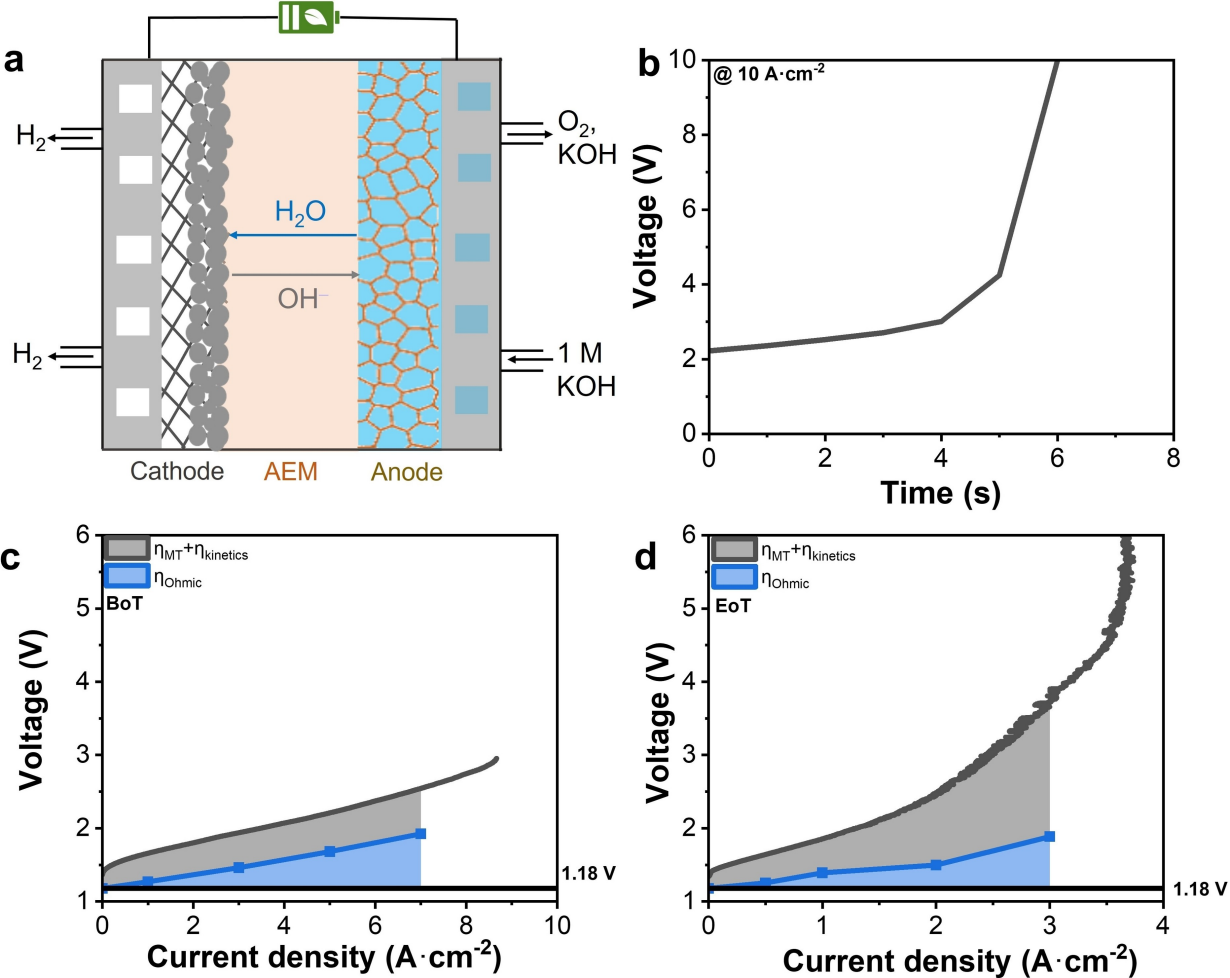
Schematic illustration of AEMWE and stability test of a current benchmark. a, Schematic illustration of AEMWE. b, Stability test of the benchmark at 10 A ⋅ cm^−2^. c, Voltage breakdown of the benchmark at BoT. d, Voltage breakdown of the benchmark at EoT. The benchmark: NiFe/NF∥Sustainion∥Pt/C. Cell temperature at 80 °C, 1 M KOH as electrolyte. 1.18 V is the thermodynamic voltage of water electrolysis at 80 °C.

Anion exchange membrane water electrolyzer (AEMWE) (Figure 1a) is recognized as a cost‐effective alternative to proton exchange membrane water electrolyzer (PEMWE) for green hydrogen production because the alkaline environment allows the use of low‐cost hardware and catalysts.[^1^, ^2^, ^3^, ^4^, ^5^] The cost of stack accounts for approximately 60 % of the total capital cost (CAPEX) of AEMWE.[^2^, ^5^] Intense research is being done to develop new materials, such as membranes, ionomers, and catalysts, as well as new device designs, in order to reduce this capital cost.[^1^, ^2^, ^3^, ^4^, ^5^] A perhaps more effective, but often neglected, approach to reduce CAPEX is to operate AEMWE at a high current density. This approach achieves a high H_2_ production rate per unit to reduce largely the stack footprint, volume, and materials use, and hence the CAPEX. A recent analysis shows that the CAPEX of PEMWEs exponentially decreases with the current density, whereas the operational cost (OPEX) is a function of the operating current density j via U_cell_ and depends on the current–voltage characteristics. The cost increases logarithmically at low current density and then linearly at higher current densities.^[6]^ These two opposite trends lead to an optimal current density for the total cost of hydrogen production (CAPEX+OPEX). Whereas the current optimum is about 2.5 A ⋅ cm^−2^, with the decrease of renewable electricity cost, the optimum current density increases towards the 10 A ⋅ cm^−2^ range.[^6^, ^7^] Due to the similarity of AEMWE and PEMWE in cell design and operating principles,[^2^, ^8^] we show that a similar scenario is valid for AEMWE (Supporting Information).

Current state‐of‐the‐art AEMWEs are normally operated (in electrolysis mode) at current densities below 3 A ⋅ cm^−2^.[^1^, ^2^, ^3^, ^4^, ^5^] Operating a water electrolyzer at a current density higher than 3 A ⋅ cm^−2^ is expected to pose challenges in both materials and the balance of plant, including particularly the performance and durability of membranes, ionomers, and catalysts.[^1^, ^2^, ^3^, ^4^, ^5^] A few recent reports started to describe AEMWE performance at high current densities.[^9^, ^10^, ^11^, ^12^, ^13^] For example, Li et al. reported an AEMWE with a polyphenylene‐based anion exchange membrane (AEM), a Ni−Fe‐based anode, and a Pt−Ru/C‐based cathode that reached 5.5 A ⋅ cm^−2^ at 1.85 V at 85 °C.^[9]^ Hu et al. reported an AEMWE with a poly(aryl‐ piperidinium)‐based AEM, a Pt−Ru/C‐based cathode, and an IrO_2_ anode, that reached 16 A ⋅ cm^−2^ at 2.0 V at 80 °C.^[10]^ Hu et al. also reported an analogous system with a new AEM that reached 13 A ⋅ cm^−2^ at 2.0 V at 80 °C.^[11]^ However, these data were only obtained in transient measurements, i.e., by linear sweep voltammetry (LSV). A sustained, steady‐state operation of an AEMWE at a current density greater than 3 A ⋅ cm^−2^ has not been reported. As we show below, an AEMWE with typical materials has an operational lifetime of only seconds at 10 A ⋅ cm^−2^, which might explain the dearth of their reported operation at a current density greater than 3 A ⋅ cm^−2^.

In this work, we uncover the AEM degradation and severe increase of mass transport resistance as the main causes of the failure of stable AEMWE operation under an ultrahigh current density (10 A ⋅ cm^−2^). By using an efficient AEM, ionomers, and catalysts, as well as by optimizing the catalyst‐porous transport layer (PTL) interface, we greatly improve the gas, liquid, and ion transport at the three‐phase interface of AEMWEs. As a result, we achieved an AEMWE that can stably operate for more than 800 hours at 10 A ⋅ cm^−2^. This operational lifetime is a 5‐order of magnetite improvement over a current benchmark.

## Results and Discussion

### Stability of a Benchmark AEMWE

**Figure 2:**
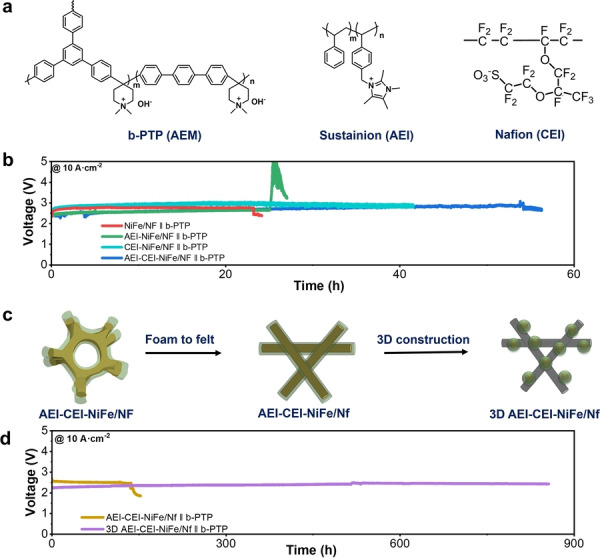
Boosting AEMWE stability at 10 A ⋅ cm^−2^. a, Molecular structures of AEM: branched poly(terphenyl piperidinium) (b‐PTP), AEI: Sustainion XB‐7, and CEI: Nafion. b, Stability tests upon changing membrane and ionomers at 10 A ⋅ cm^−2^. c, Schematic illustration of different catalyst‐PTL interfaces. d, Stability tests upon changing catalyst‐PTL interfaces at 10 A ⋅ cm^−2^. Coupled with Pt/C as cathode. Cell temperature at 80 °C, 1 M KOH as electrolyte.

To probe the performance of AEMWEs at ultrahigh current densities, we assembled a benchmark AEMWE using a representative cell configuration. A self‐supported Ni−Fe oxyhydroxide on Ni foam (NiFe/NF; NF=nickel foam) was used as the anode, a commercial Sustainion X37–50 (grade RT) AEM was used as the membrane, and a Pt/C (weight fraction of 40 % Pt) on carbon fiber was used as the cathode (Figure 1a, also see Supplementary Methods).[^1^, ^2^, ^3^, ^4^, ^5^, ^14^, ^15^] Note that the electrode materials are made of typical catalysts such as NiFe oxyhydroxide and Pt/C, and the porous transport layer materails such as nickel foam and carbon paper are commercially available. The detailed characterizations of the NiFe/NF anode were displayed in Figure S1. Here, the lifetime is defined as the time when a sudden potential surge appeared and/or the Faradaic efficiency (FE) for H_2_ production is lower than 100 %. The operational lifetime of this AEMWE was only about 6 seconds at 10 A ⋅ cm^−2^, after which the cell potential suddenly surged from 2.3 to 10 V (Figure 1b). Figures 2c and 2d show the transient LSV curves at the beginning of test (BoT) and the end of test (EoT), respectively. In comparison to BoT, EoT experienced a significant increase in cell voltage at the same current density (Figures 1c and 1d). For example, at 3 A ⋅ cm^−2^, the cell voltage increased from 1.92 V at BoT to 3.69 V at EoT.

We attempted to use electrochemical impedance spectroscopy (EIS) measurements to investigate the failure mechanisms of this AEMWE, encompassing the simulation of Ohmic, charge‐transfer and mass‐transfer resistances. Here the Ohmic resistance includes the ionic resistance of AEM, charge‐transfer resistance reflects the kinetics of hydrogen evolution reaction (HER) at the cathode and oxygen evolution reaction (OER) at the anode, whereas the mass‐transfer resistance includes the resistance due to OH^−^ ion, water, and gas transport at the PTL‐catalyst‐membrane interfaces. However, the EIS data suggests the existence of multiple semi‐circles that are hard to model, and at high current densities, the data showed severe oscillation at low frequencies. Thus, we were only able to abstract Ohmic resistances from the EIS data. Consequently, we could only break the total voltage into the contributions from ohmic resistance and the rest (Figure 1c and 1d).

EIS results indicated a substantial increase in Ohmic resistance from 0.09 Ω ⋅ cm^2^ at BoT to 0.23 Ω ⋅ cm^2^ at EoT at 3 A ⋅ cm^−2^ (Supporting Information Table 1). By multiplying this Ohmic resistance increment by the current density, an Ohmic overpotential increment of 0.42 V at 3 A ⋅ cm^−2^ was determined, accounting for about 1/4 of the total voltage increase at this current density. After the test, the membrane sample had poor solubility in DMSO, likely due to crosslinking during operation. NMR analysis of the part of the AEM that was soluble after stability test (Supplementary Figures 2 and 3) revealed that while the backbone of the membrane remained stable, the ionic imidazole groups had undergone ring‐opening decomposition, leading to an ionic loss of about 66 % in 6 seconds. The ring opening of imidazolium yields an amide intermediate that is possible source for cross‐linking (Figure S2). Thus, the Sustainion X37–50 (grade RT) AEM seemed to undergo degradation, which was the origin of a higher Ohmic resistance. The remaining 3/4 of the total voltage increase was due to the charge and mass transfer overpotentials. This increase might be a result of membrane degradation which affected the catalyst‐membrane interface, but the details are unclear as we could not abstract charge and mass transfer resistances. The above data indicates that the failure of the cell at ultrahigh current densities resulted from, at least partially, the membrane degradation.

### Boosting AEMWE Stability at 10 A ⋅ cm^−2^

To improve the operational stability of AEMWEs at ultrahigh current densities, we decided to change the AEM from the commercial Sustainion to our Branion b‐PTP AEM (Figure 2a) from NovaMea that had proven to be highly robust in AEMWEs in recent reports.[^16^, ^17^, ^18^] Amazingly, the usage of b‐PTP AEM improved the lifetime from 6 seconds to 23 hours at 10 A ⋅ cm‐2 compared to that of Sustainion AEM, which was a 13,800‐fold improvement (Figure 2b). Having an AEM that is highly conductive and robust at ultrahigh current densities such as b‐PTP, thus, is a prerequisite for the stable operation of AEMWEs at such current densities. This improvement set the stage for further optimization as described below. Post‐stability NMR analysis indicates that the polymer backbone remained stable during operation, while an about 22 % ionic loss due to the decomposition of the piperidinium ionic groups occurred (Supplementary Figures 4 and 5). The much lower ionic loss of b‐PTP after 23 hours compared to that of Sustainion after 6 seconds indicates the robustness of the b‐PTP AEM under ultrahigh current densities.

The stable operating cell voltage of the above system is 2.76 V at 10 A ⋅ cm^−2^ (anode: NiFe/NF). We sought to increase the performance of the cell by improving the mass transport which could become limiting at ultrahigh current densities. As the anode was devoid of an ionomer, we decided to add an anion exchange ionomer (AEI) to the anode to facilitate OH^−^ transport in the catalyst layer (NiFe/NF→AEI‐NiFe/NF). Gratifyingly, the addition of Sustainion XB‐7 (Figure 2a, ion exchange capacity (IEC)=2.2 mmol/g) as an AEI on NiFe/NF resulted in 0.12 V reduction in cell voltage (from 2.76 to 2.64 V, Figure 2b) while the operational lifetime slightly increased from 23 to 25 h. Nafion is a typical cation exchange ionomer (CEI; Figure 2a), but it can serve as a stabilizing, connecting and binding agent for the catalysts as well.[^19^, ^20^] Due to the presence of perfluoroalkyl backbones in Nafion, it creates a highly hydrophobic environment affecting local water management.[^21^, ^22^] Li et at. found that the ionic‐group‐sparse domains in Nafion ionomer create hydrophobic interface and reactant transport channels with lower water content, accordingly, its surface hydrophobicity enhance the concentration‐polarized regions of the fuel cell polarization curve.^[21]^ Jervis et al. also reported that electrode with Nafion yields better electrodes, compared to AEI which with large catalyst agglomerates, leading to a poorer accessibility to active sites and lower limiting HOR current.^[23]^ Osmieri et al. reported that the combined used of AEI (PiperION) and Nafion gives structural stability to the catalyst, thanks to the superior binding abilities of Nafion.^[24]^ We thought Nafion might enhance the adhesion of the catalyst layer. Thus, we added a small amount of Nafion (see Supplementary Methods) to the anode to stabilize the catalyst layer (NiFe/NF→CEI‐NiFe/NF). The addition improved the lifetime to 41 h (Figure 2b). We also used PTFE (Polytetrafluoroethylene) instead of Nafion as the binder, but the operational lifetime was only 13 h. Fell of the catalyst from the electrode was observed. This result indicates that PTFE is not as good as a binder as Nafion. In the configuration of Nafion‐NiFe/NF, the cell voltage increased compared to the cell with no ionomer. This result likely originated from the anionic charge of the Nafion polymer, which impeded OH^−^ transfer. To combine the advantages of both AEI and CEI, we added both components (NiFe/NF→AEI‐CEI‐NiFe/NF). Gratifyingly, this resulted in a cell with both a lower voltage (from 2.76 to 2.67 V) and longer stability (54 h).

The PTLs are essential components in AEMWE, and they may influence all components of cell over‐voltage including kinetic, Ohmic, and mass transport resistance.[^25^, ^26^] Most current AEMWEs use Ni foam as the anode PTL due to its high stability, porosity, and low cost,[^1^, ^2^, ^3^, ^4^, ^5^] but the surface of Ni foam is relatively rough and might damage the MEA upon long‐term operation. Compared to Ni foam, Ni felt (Nf) is also highly porous and even has a higher electrochemical surface area (ECSA, Figure S6), and a softer surface. The latter properties might be beneficial for the stability of MEA devices. Indeed, by using Nf as PTL and otherwise identical components and configurations (AEI‐CEI‐NiFe/NF→AEI‐CEI‐NiFe/Nf, Figure 2c), we further increased the operational stability from 50 h to over 140 h (Figure 2d). The cell voltage remained similar at about 2.54 V, suggesting that ECSA was not a limiting factor in these devices.

Exploring further, we then developed a three‐dimensional (3D) anode comprising NiFe powders and ionomers, spray‐cast onto the Ni felt substrate, thereby forming a 3D morphology with catalyst and ionomer percolation paths (AEI‐CEI‐NiFe/Nf→3D AEI‐CEI‐NiFe/Nf, Figure 2c). Such an anode maximizes the three‐phase reaction interface across an extended 3D morphology. To our delight, the resulting AEMWE system delivered 10 A ⋅ cm^−2^ at only ~2.3 V, which was 0.24 V further lower than the analogous system without the 3D anode (Figure 2d). This result indicates that the 3D morphology is beneficial for charge and mass transfer. Importantly, this AEMWE system exhibits an exceptional lifetime of 850 h at this ultrahigh current density (Figure 2d). Post‐modem analysis of the electrodes showed that whereas the AEMWEs with AEI‐CEI‐NiFe/NF and AEI‐CEI‐NiFe/Nf suffered from some catalyst loss in the anode, the catalysts remained adhered on the electrodes in the AEMWE with the 3D CEI‐AEI‐NiFe/Nf anode (Figure S7). These results indicate that the 3D structure also enhances the interfacial adhesion between the catalysts and PTLs.

We have also measured the transient AEMWE performance of AEMWEs with all the configurations described above using LSV. The I–V data are shown in Figure S8. For the best AEMWE (with the 3D AEI‐CEI‐NiFe/Nf anode), the cell voltage was merely 1.95 V at 10 A ⋅ cm^−2^, comparable with the best transient data in the literature.[^7^, ^8^, ^9^, ^10^, ^11^] The low cell voltage likely contributes to the electrolyzer's exceptional lifetime at ultrahigh current densities. Note that the cell voltages in the transient LSV measurements are all substantially lower than in steady‐state electrolysis (Supporting Information Table 2, SI), highlighting the “non‐operational” nature of the LSV data.

An accelerated stress testing (AST) was conducted for the AEMWE with the 3D CEI‐AEI‐NiFe/Nf anode. A square wave potential cycling between 1.45–2 V was applied with a 5–10 s hold at each potential. The LSV data (Figure S9) showed a small performance deterioration after AST.

We tested our best AEMWE (with the 3D CEI‐AEI‐NiFe/Nf anode) in pure‐water‐fed mode. The LSV data (Figure S10) showed that the cell voltages are much higher than in the 1 M KOH‐fed systems (Figure S8). This result is consistent with our previous work showing the challenges in mass transport for pure‐water‐fed AEMWE.^[16]^

### Analysis

**Figure 3:**
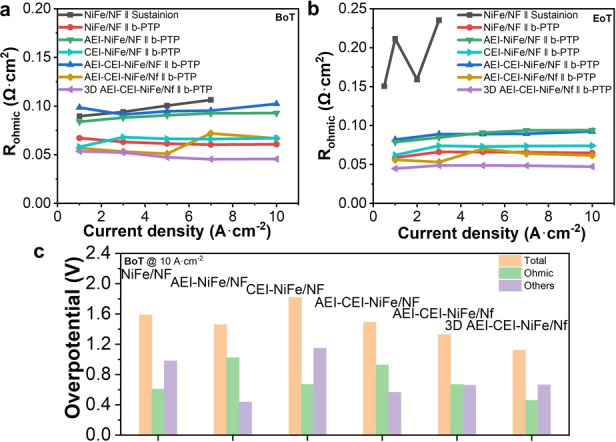
Analysis of factors contributing to cell performance. a, Ohmic resistance at BoT. b, Ohmic resistance at EoT. c, Overpotential contributions at 10 A ⋅ cm^−2^ and BoT in Figure 2.

The EIS data of all relevant cells are becoming difficult to fit at increasing current density (see discussion above; Figure S11), so we were only able to abstract the ohmic resistances. Figure 3a and b show the ohmic resistances as a function of current density. Among all configurations, only the cell with the Sustainion X37–50 (grade RT) AEM suffered from a large increase in ohmic resistance at EoT compared to at BoT (Figure 3a and b). The ohmic resistances are largely the same at different current densities for the other cells that used the b‐PTP AEM. These results indicate that the b‐PTP AEMs used in the other cells remain stable during operation. The ohmic resistance of the cell with a b‐PTP AEM is lower than that with a Sustainion AEM under otherwise the same configurations (compare NiFe/NF∥b‐PTP to NiFe/NF∥Sustainion), indicating that b‐PTP AEM is more conductive. The difference gets bigger at higher current densities. Thus, the lower performance of Sustainion‐containing cells can be, at least partially, attributed to their higher Ohmic loss.

The total cell voltages at 10 A ⋅ cm^−2^ were then broken into contributions from ohmic resistance and the rest (including charge transfer and mass transport) (Figure 3c). The addition of either AEI or CEI slightly increased ohmic resistances due to the covering of some active sites in catalysts. Meanwhile, AEI facilitated OH^−^ transport, thereby reducing mass transfer resistances (compare AEI‐NiFe/NF to NiFe/NF). On the other hand, CEI enhanced catalyst layer binding, thus increasing cell stability. CEI, however, impeded OH^−^ transport and increased the cell voltage (compare CEI‐NiFe/NF to NiFe/NF). Fortuitously, the simultaneous addition of AEI and CEI combined their beneficial effects while avoiding their shortcomings (compare AEI‐CEI‐NiFe/NF to NiFe/NF).

Going from Ni foam to Ni felt PTL(compare AEI‐CEI‐NiFe/Nf to AEI‐CEI‐NiFe/NF), the ohmic resistance was reduced, suggesting a better interfacial contact. The total cell voltage remained the same, which suggests that either charge or mass transfer resistance was increased. It could be that Ni felt is a worse support than Ni foam for the self‐supported NiFe OER catalyst. Changing the self‐supported catalyst to a powdery catalyst for the Ni felt further lowered the ohmic resistance (compare 3D AEI‐CEI‐NiFe/Nf to AEI‐CEI‐NiFe/Nf), suggesting a better interfacial contact among the AEM, catalyst, and PTL. The decrease in ohmic resistance is the main contributor (Δ=0.21 V at 10 A ⋅ cm^−2^) for the total voltage decrease (0.24 V). It is noted that the sum of charge and mass transfer overpotential for the overall best cell (3D AEI‐CEI‐NiFe/Nf∥b‐PTP) is almost 0.66 V higher than that of the cell AEI‐NiFe/NF∥b‐PTP. Thus, there is further room for improvement for the best‐performing cell.

During our optimization, we have changed parameters related to AEM, AEI, CEI, PTL, and 3D structure. Our analysis reveals their different influences. The stability of AEM is the most important factor for the stable operation of AEMWE at ultrahigh current density. An appropriate binder (e.g., CEI), PTL, as well as electrode‐catalyst interface are also beneficial for the stability. In terms of energy efficiency, the optimized electrode‐catalyst interface from the 3D structure appears to be the most advantageous.

### Comparison to the State‐of‐the‐art

**Figure 4:**
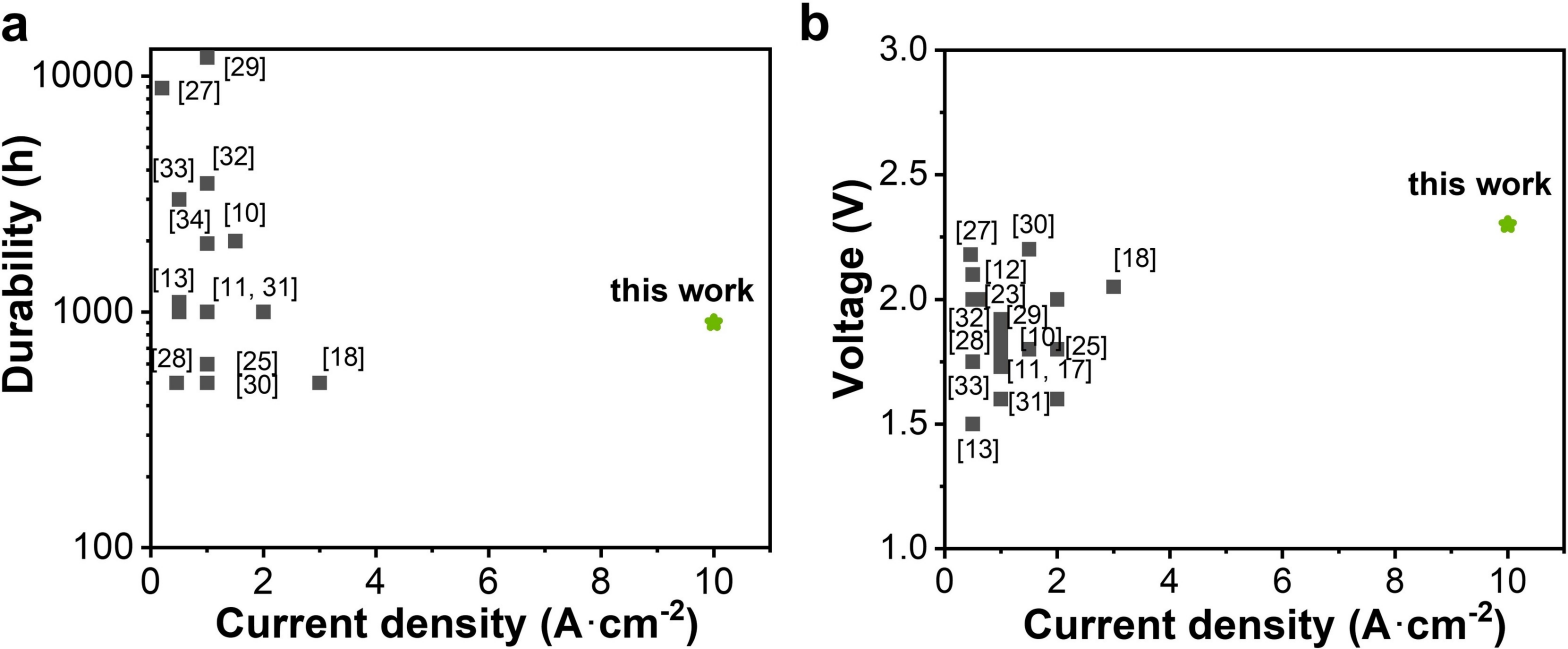
Comparison to state‐of‐the‐art AEMWEs. a, Lifetime vs. current density. b, Voltage during stability test vs. current density.

We have compared the AEMWE performances of our system with state‐of‐the‐art systems reported so far (Figure 4).[^10^, ^11^, ^13^, ^18^, ^25^, ^27^, ^28^, ^29^, ^30^, ^31^, ^32^, ^33^, ^34^] Current durability tests of AEMWEs are typically done under a normal current density (<3 A ⋅ cm^−2^) with lifetimes of around 1000 h or lower (Figure 4a). The steady‐state cell voltages of state‐of‐the‐art examples are typically in the range of 2±0.3 V at current densities of 1–3 A ⋅ cm^−2^ (Figure 4a). Remarkably, our system demonstrates durable AEMWE operation of more than 800 h at an ultrahigh current density of 10 A ⋅ cm^−2^, while maintaining a comparable cell voltage to current low‐current‐density systems (Figure 4b).

## Conclusion

In summary, by using a highly conductive and robust AEM, we were able to obtain AEMWEs that operate at an ultrahigh current density of 10 A ⋅ cm^−2^ with extended lifetimes. Through a systematic optimization to improve interfacial contact and OH^−^ transfer, we achieved an AEMWE that stably operates at 10 A ⋅ cm^−2^ for more than 800 hours, which is more than 500’000 times higher than the benchmark example. The cell voltage is only about 2.3 V at this ultrahigh current density, which is comparable to the voltage of state‐of‐the‐art devices operating at below 3 A ⋅ cm^−2^. Our analysis shows that charge and mass transfer resistances are not yet optimal for this cell. Future work should be aimed at alleviating these resistances. This work lays the groundwork for developing ultrahigh current density AEMWEs. The work also highlights the challenges of designing such AEMWEs, including highly conductive and stable AEMs, efficient and robust catalyst layers, and facile interfacial mass transfer.

## Supporting Information

The authors have cited additional references within the Supporting Information.[^35^, ^36^, ^37^]

## Conflict of Interests

The PTP AEM and PBP ionomer used in this work are commercialized by an EPFL Startup NovaMea SA, of which X.H. has a financial interest.

1

## Supporting information

As a service to our authors and readers, this journal provides supporting information supplied by the authors. Such materials are peer reviewed and may be re‐organized for online delivery, but are not copy‐edited or typeset. Technical support issues arising from supporting information (other than missing files) should be addressed to the authors.

Supporting Information

## Data Availability

The data that support the findings of this study are available in the supplementary material of this article.
