# Supplementary material for: Anion Exchange Membrane Water Electrolysis at 10 A ⋅ cm−2 Over 800 Hours
Source: Angew Chem Int Ed Engl. 2024 Nov 7;64(1):e202413698. doi: 10.1002/anie.202413698 (PMC11701342; doi:10.1002/anie.202413698)
Supplement: Supplementary file 1 — Supporting Information [file ANIE-64-e202413698-s001.pdf]

Angewandte Chemie
Eine Zeitschrift der Gesellschaft Deutscher Chemiker

Supporting Information

Anion Exchange Membrane Water Electrolysis at 10 A·cm^{-2} Over 800 Hours

*Y. Zheng, W. Ma, A. Serban, A. Allushi, X. Hu**

Supplementary methods

Cost Analysis

A cost analysis is performed to identify the most appropriate operating current density for AEMWE. Both cost and technical aspects have been taken into consideration. There are two major cost contributions to the hydrogen cost by water electrolysis: (i) the capital expenditure (CAPEX) that takes into account the total investment cost (the electrolysis stack but also all ancillary equipment, including water purification, AC/DC conversion, and gas treatment units) and the lifetime of this investment; (ii) the operational expenditure (OPEX) that takes into account the energy cost and maintenance costs.[1,2]

Assuming that lifetime of operation is not affected by the operating current density, a straightforward mass balance equation yields the expression of the CAPEX contribution to the hydrogen cost ($/kg$_{H_2}$):

$$CAPEX = (\frac{2F \cdot IC}{T \cdot S \cdot M_{H_2}}) \cdot \frac{1}{j}$$

Where, IC is initial investment cost; T is lifetime of the electrolysis plant (in s), S is the active surface area, M is H_2 molecular weight, F is faraday constant, j is current density.

Assuming that water consumption and the energy consumption of ancillary equipment and water purification can be neglected (5-10%), compared to the electricity consumption of the electrolysis stack (90-95 %), a mass balance equation yields the expression of the OPEX contribution ($/kg$H_2$):

$$OPEX = \frac{U_{cell} \cdot F}{3600(J \cdot kg \cdot kWh^{-1} \cdot mol^{-1})} \cdot EC$$

Where EC is electricity cost, U_{cell} is cell voltage, $(J \cdot kg \cdot kWh^{-1} \cdot mol^{-1})$ is unit.

$$U_{cell}(j) = E_{rev}(P,T) + j\sum_{i=1}^{n} r_i + |\eta_{H_2}(T,P)| + \eta_{O_2}(T,P)$$

E_{rev} is the reversible voltage, r_i is the surface resistance of the ith cell component, η_{H_2} is the absolute value of the HER overvoltage. η_{O_2} is the OER overvoltage. Thus, the OPEX is a function of the operating current density j via U_{cell} and depends on the current–voltage characteristics. The cost increases logarithmically at low current density and then linearly at higher current densities.

Electrode Preparation

Both self-supported and powdery NiFe OER catalysts were synthesized following the same procedure.[3] Typically, $Ni(NO_3)_2 \cdot 6H_2O$ (2.0 mmol), $Fe(NO_3)_3 \cdot 9H_2O$ (0.5 mmol) were dissolved in H_2O with vigorous stirring, then NH_4F (10 mmol) and urea (25 mmol) were added. The mixed solution was stirred for 30 mins and then transferred to a Teflon-lined stainless-steel autoclave. The autoclave was heated at 120 °C for 16 h. After cooling down to room temperature, the yellowish solid was washed 3 times by DI water and ethanol, and then naturally dried. The preparation of self-supported NiFe-based catalyst was putting different Ni substrates (300 μm Ni felt and foam, from Dioxide Materials and Fiaxell, respectively) into solution above in autoclave. They are denoted as NiFe/Nf and NiFe/NF, respectively. The catalyst loadings on self-supported NiFeLDH were 20 $mg \cdot cm^{-2}$. AEI coated self-supported NiFe LDH Ni foam was denoted as AEI-NiFe/NF and had 20 % AEI. CEI coated self-supported NiFe/NF was denoted as CEI-NiFe/NF and had 3 % CEI. AEI and Nafion coated self-supported NiFeLDH Ni foam/felt were denoted as AEI-CEI-NiFe/NF and AEI-CEI-NiFe/Nf, respectively and had 20 % AEI and 3 % Nafion. AEI and Nafion solution were mixed and sonicated in ice bath for at least 30 min then sprayed onto the catalyst substrates. The Nickel foam was compressed from 1.6 mm to 300 μm by hot pressing before depositing the catalyst in order to improve the smoothness and flatness of its surface and ensure the same thickness between electrodes, which are important for the assembly of the AEMWE.

Powdery NiFe LDH was made into catalyst ink and then sprayed onto the bare Ni felt. Normally, Sustainion XB-7 (Figure 2a, ion exchange capacity IEC = 2.2 $mmol \cdot g^{-1}$) as AEI was added at 20 %, Nafion 117 was added at 3 %, and PTFE powder was always kept at 8 % of the mass fraction in the catalyst layer. The catalyst was denoted as 3D AEI-CEI-NiFe/Nf and its loading was maintained at 3 $mg \cdot cm^{-2}$.

Cathode preparation is done by the same CCS method, using 20 % NovaMea's branched poly(biphenyl piperidinium) ionomer (b-PBP) with IEC of 3.1 $mmol \cdot g^{-1}$ (as AEI) and 1.6 % PTFE powder. The cathode catalyst was Pt (weight fraction of 40 %) supported on Vulcan XC-72R (Alfa Aesar HiSPEC 4000). The ink dispersions were sprayed onto Toray TGP-H-060 PTLs with PTFE wetproofing (weight fraction of 5 %). The catalyst loadings were kept at 0.5 mg_{pt}/cm^2.

AEMWE Assembly and Break-in Procedure

25 μm NovaMea's branched poly(terphenyl piperidinium) (b-PTP) AEM with 2.5 mol% of 1,3,5-triphenylbenzene comonomer (IEC = 2.81 $mmol\cdot g^{-1}$) was tested in the study (Fig. 2a). Electrodes that contained ionomer and AEMs were soaked in separate 1 M aqueous KOH solutions overnight. Before cell assembly, the electrodes and AEMs were soaked in fresh 1 M KOH solution for 60 min, exchanging the solution twice during this time. After soaking, electrodes and AEMs were pressed together in the cell to form MEAs. The MEAs were loaded into Scribner hardware between the single-pass serpentine flow of Ni plate in anode and graphite plate in cathode with an active area of 1 cm^2 due to the limitation of potentiostat's current range. The MEA was sealed and compressed with 12 mil (305 μm) and 5 mil (127 μm) PTFE gaskets for anode and cathode, respectively, at 2.5 N·m torque. Cell temperature was controlled by a home-assembled rod heater and sensor at 80 °C. The electrolyte of 1 M KOH solution flow rate was controlled by a peristaltic pump at 1 mL/min and fed into anode, recirculated for the duration of the experiment.

Cell performance was analyzed through linear sweep voltammetry (LSV), current hold, and electrochemical impedance spectroscopy (EIS) measurements. Galvano-EIS over a frequency range from 0.01 Hz to 100 kHz was performed by Autolab PGSTAT302N at 0.5, 1, 2, 3, 5, 7, 10 $A\cdot cm^{-2}$. LSV curves were taken at a scan rate of 50 $mV\cdot s^{-1}$ until 10 $A\cdot cm^{-2}$ was reached. The break-in process was conducted at a scan rate of 50 $mV\cdot s^{-1}$ between 1.3 to 2 V. After the break-in procedure, the beginning of the test (BoT) was set. LSV and EIS measurements were taken at the beginning and end of cell test (BoT and EoT). Ohmic resistances were extracted from EIS analysis. The exiting hydrogen volumetric flowrate was measured by collecting gas over water in graduated cylinder during operation at 10 $A\cdot cm^{-2}$. The flowrates measured during stability test were among 78.5 – 80 $ml\cdot min^{-1}$ which correspond to 97.6 - 99.5 % faradaic efficiency (FE). Once the FE was lower than 97 %, the end of test (EoT) was set.

Electrochemical Test

Three-electrode electrochemical tests were performed in 1 M KOH electrolyte (pH = 14). The working electrode was the as-prepared catalyst, and the Ag/AgCl and Pt wire were selected as the reference and reversible hydrogen electrode (RHE). Electrochemical active surface area (ECSA) measurement followed the same procedure in our previous publication.[4]

Characterizations

X-ray diffraction (XRD) patterns were conducted on an ANalytical Aeris diffractometer using Cu K_α radiation (40 kV, 15 mA). Scanning electron microscopy (SEM) images were conducted on a Zeiss GeminiSEM 300 scanning electron microscope at 3 kV. Transmission electron microscopy (TEM) measurements were conducted on an FEI Talos F200S electron microscope at 200 kV.

Polymer Characterization

^{1}H NMR spectra of the Sustainion and b-PTP AEMs at BoT and EoT were analyzed by a Bruker DRX400 spectrometer at 400.13 MHz using DMSO-d6 (δ = 2.50 ppm). 5 – 10 drops of TFA were then added to protonate the tertiary amine groups and to shift the water peak from 3.5 ppm to above 10 ppm (downfield region). Aromatic signals appeared in the region 6.5 - 8 ppm in the ^{1}H NMR spectra. There is usually no degradation in the aromatic region during the test. At EoT, the membrane samples were washed 3 times by ethanol to get rid of contaminated catalysts then ion-exchanged to Br form by immersing them in 1 M NaBr solution for 3 days. During this period, the 1 M NaBr solution was replaced three times. Subsequently, the membranes were washed with deionized water to remove any low molecular weight degradation product generated during the test. Before dissolving in the DMSO, the sample was dried at 80 °C overnight. The dissolved sample in DMSO was ready for measurement after filtering (Ø = 13 mm, PTFE membrane, pore size 0.2 μm).[5]

Accelerated Stress Testing (AST)

Accelerated stress testing was performed with 1 M KOH fed continuously to the anode. The cell was subjected to voltage between 2 and 1.45 V (5 s at each voltage for the first 500 cycles then 10 s for each voltage for 4500 cycles) applied in a square wave fashion. After the AST procedure, LSV was taken to monitor the degradation process.

Pure-water-fed AEMWE

AEMWE was constructed with dry cathode and deionized water in anode. The cell was operated at 80 °C and the anode electrolyte flowrate was 0.5 mL/min. The LSVs at BoT were taken.

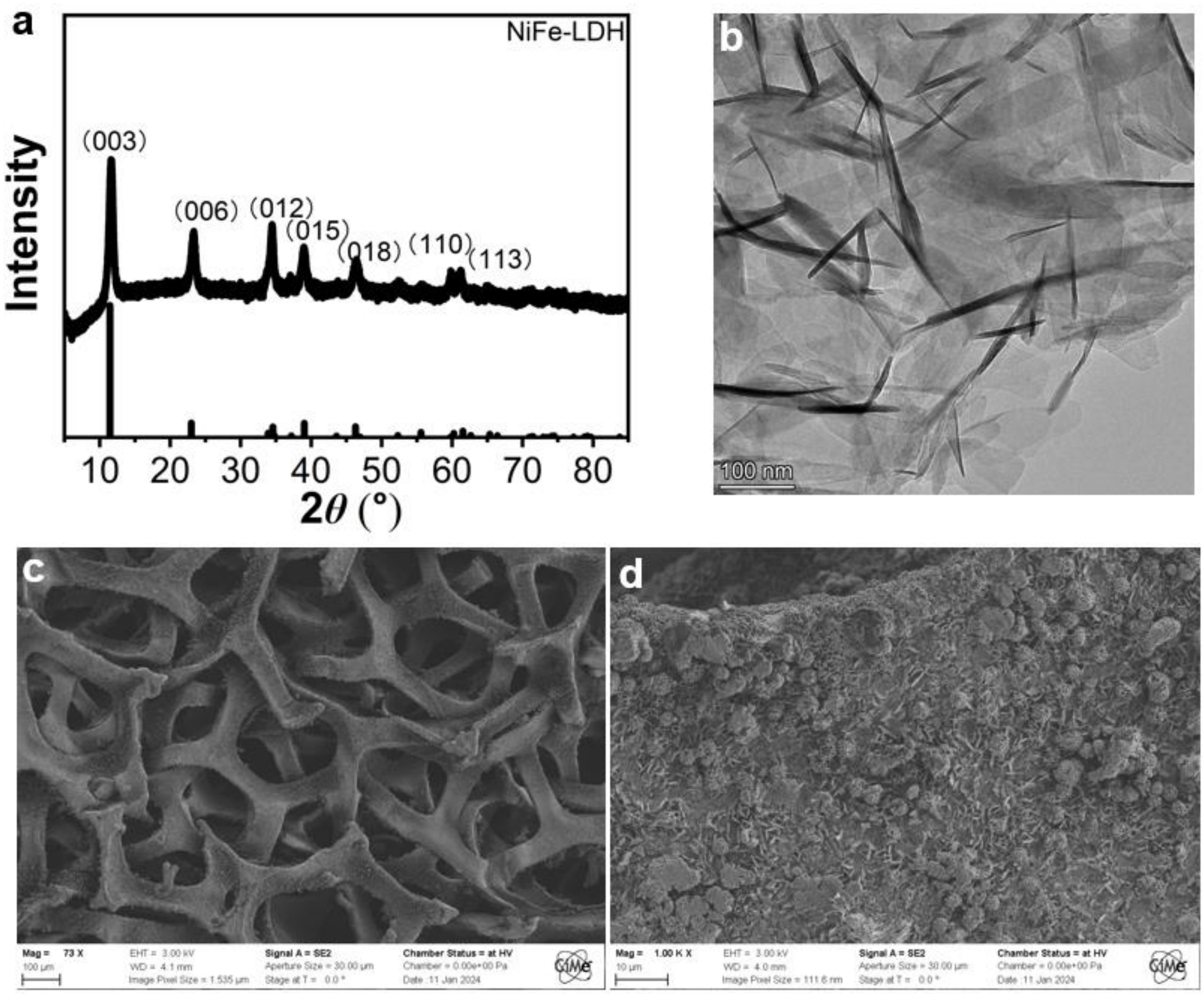

Supplementary Fig. 1│ Characterization of anode NiFe catalysts. a, Powder XRD pattern. **b**, TEM image. **c,d**, SEM images of NiFe/NF with different magnifications.

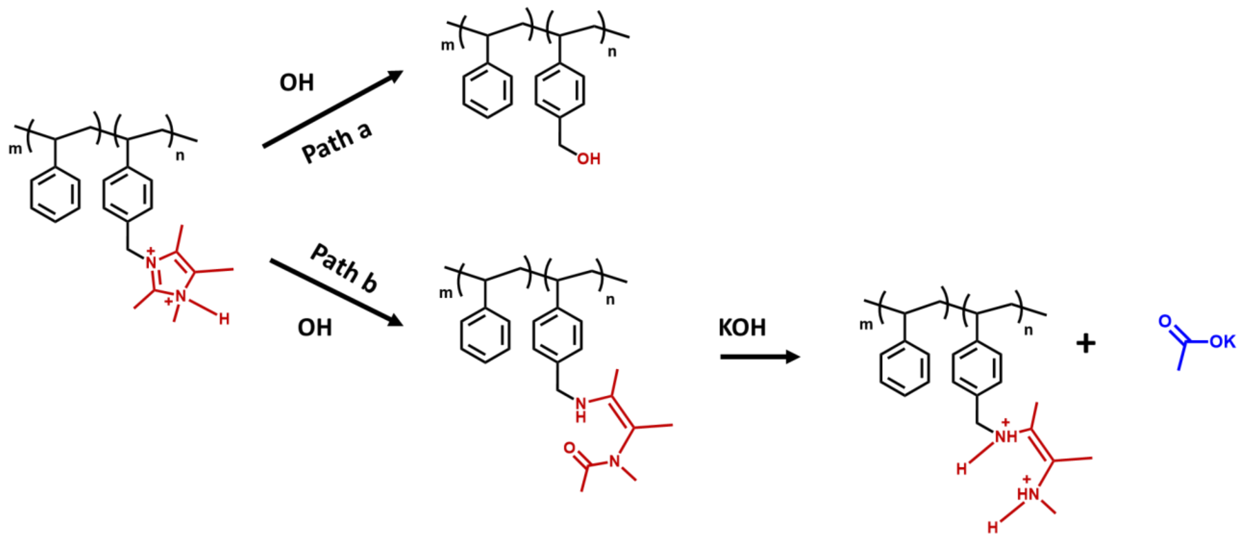

Supplementary Fig. 2 | Possible degradation pathways of Sustainion AEM.[6]

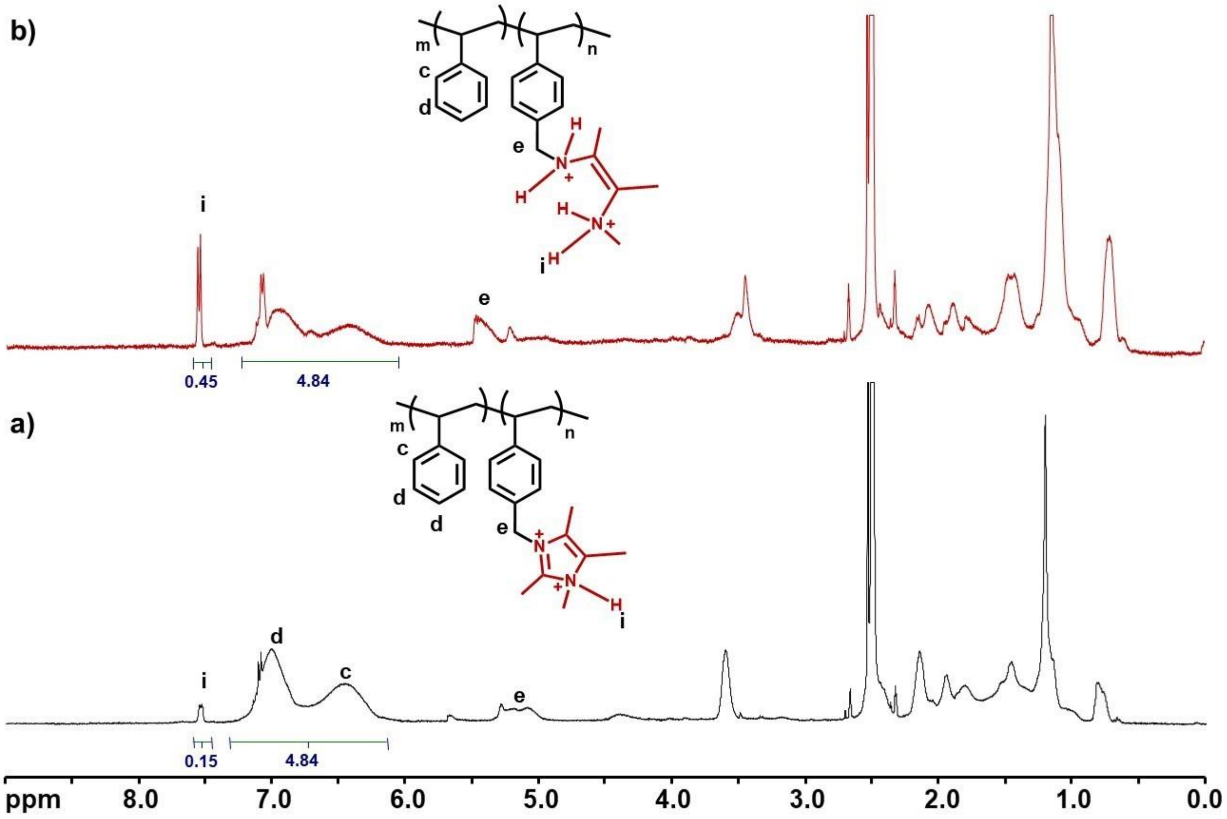

Supplementary Fig. 3 | NMR spectra of Sustainion AEM at BoT (a) and EoT (b).

Note: The commercialized Sustainion had an IEC (ion exchange capacity) ~1.2 mmol g^{-1}, which is also determined by integration and comparison of aromatic peaks and the peak from the protonated tertiary amines in the imidazolium ring ~ 7.5 ppm (i) in the HNMR spectrum (Fig. S3a). Aromatic signals appeared in the region 6.5-8 ppm in the ^{1}HNMR spectra. After the test, the membrane samples had poor solubility in the DMSO, most likely due to the crosslinking that occurred during operation. The percentage of the imidazolium ring was quantified by comparing the integrals of the relevant signal (i) with the signals in the aromatic region.

After the test, the signals in the aromatic region remain the same, indicating that there was no degradation in the aromatic region during the test. The ionic loss was estimated by integrating the corresponding ^{1}HNMR peak (the intensity of the *i* peak, Fig. S3b) and comparing it to the aromatic peaks. At EoT, the ratio of the peak *i* to aromatic peaks increased, indicating the formation of new protonated tertiary amines. This result might be due to the imidazole ring opening pathway in Fig.

S2 path b. The ionic loss was evaluated by comparing the intensity of the peak *i from* the degradation products, which correspond to two protonated secondary amines group (4 H in total) with the total signal intensity in the aromatic region, which we assumed to be unaffected by water electrolysis conditions. The ionic loss (X) is estimated to be 66%, following the equation of:

$$0.15 \times 4 \times X + 0.15 \times (1-X) = 0.45$$

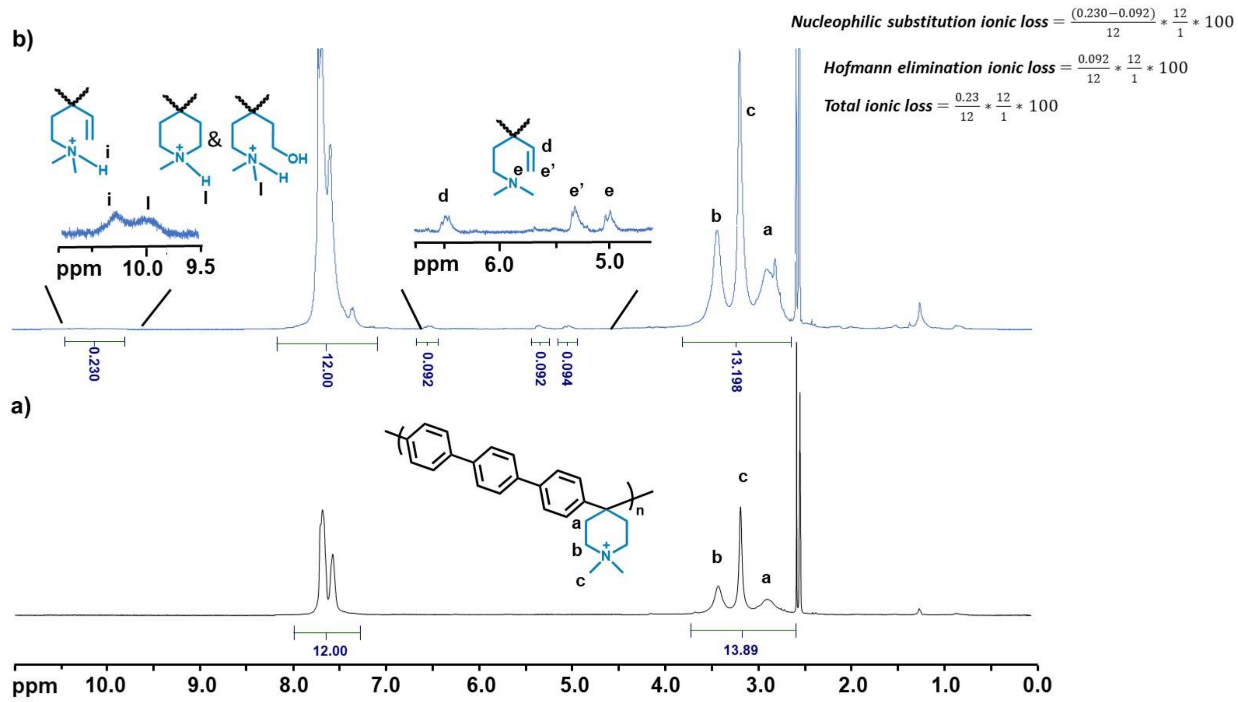

Supplementary Fig. 4│ NMR spectra of b-PTP AEM at BoT (a) and EoT (b).

Note: Aromatic signals appeared in the region 6.5-8 ppm in the ^{1}HNMR spectra. After the test, the signals in the aromatic region remain the same, indicating that there was no degradation in the aromatic region during the test. As can be seen in Fig. S4, new peaks (*a, b*) emerged in the region of 4.5-6.5 ppm. The position and the intensity of the peaks at ~ 5.0 (*e*) ~ 5.3 (*è*)~ 6.4 ppm (*d*) were 1:1:1, which suggested the presence of alkene ($=CH_2$) and alkenyl ($-CH=CH_2$) protons, respectively. The formation of the vinylic protons was probably originated from the Hofmann elimination in the piperidinium ring (Fig. S5 path a). In addition, two different peaks corresponding to protonated tertiary amines emerged at 10.3 (*i*) and 9.8 (*l*) ppm (Fig. S4b). The intensity of the former was close to the intensity of the peak *d*, and was attributed to the Hofmann elimination in the ring. The latter peak can be attributed to the methyl substitution (Fig. S5 path c) or nucleophilic ring opening substitution reaction generating hydroxyl group (Fig S5 path b).

The ionic loss was estimated by integrating the corresponding ^{1}HNMR peaks (the intensity of *d* peak) and comparing them to the aromatic signals. The ionic loss from the Hofmann elimination was estimated to be ~ 9% and the ionic loss for the methyl and nucleophilic substitution was estimated to be 13%. The total ionic loss is 22%.

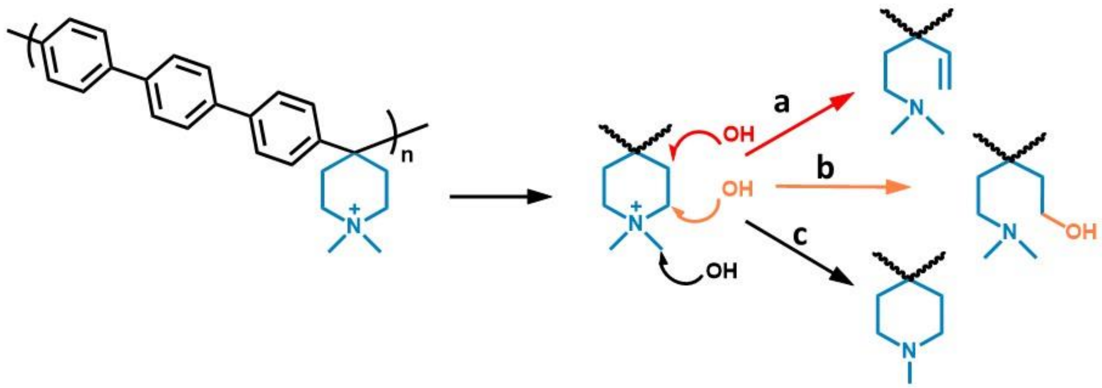

Supplementary Fig. 5│ The possible degradation pathways of b-PTP AEM: Hofmann elimination, nucleophilic ring opening substitution, and nucleophilic substitution.

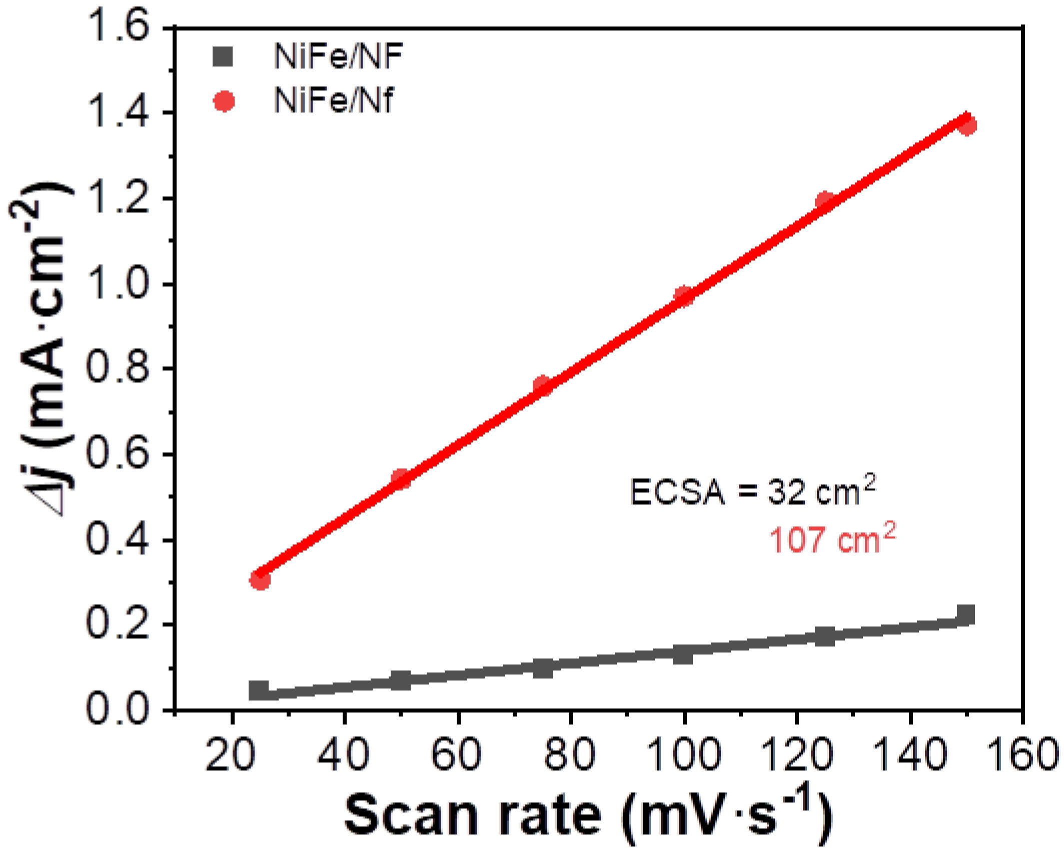

Supplementary Fig. 6 | ECSA measurements of NiFe/NF and NiFe/Nf.

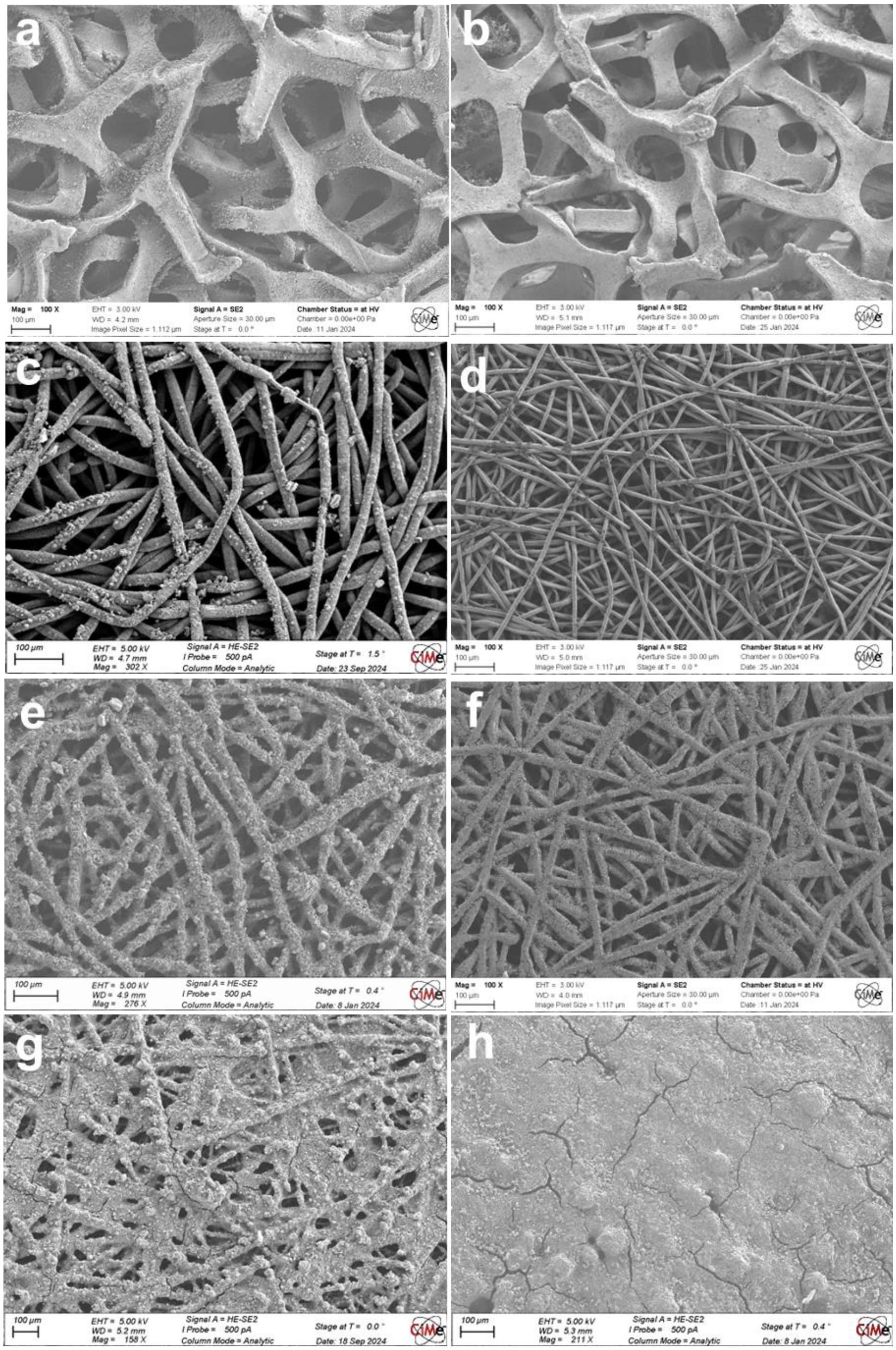

Supplementary Fig. 7│ SEM images of the electrodes at BoT and EoT. a, AEI-CEI-NiFe/NF at BoT. **b**, AEI-CEI-NiFe/NF at EoT. **c**, AEI-CEI-NiFe/Nf at BoT. **d**, AEI-CEI-NiFe/Nf at EoT. **e**, 3D AEI-CEI-NiFe/Nf at BoT. **f**, 3D AEI-CEI-NiFe/Nf at EoT. **g**, Pt/C on carbon paper at BoT. **h**, Pt/C on carbon paper at EoT.

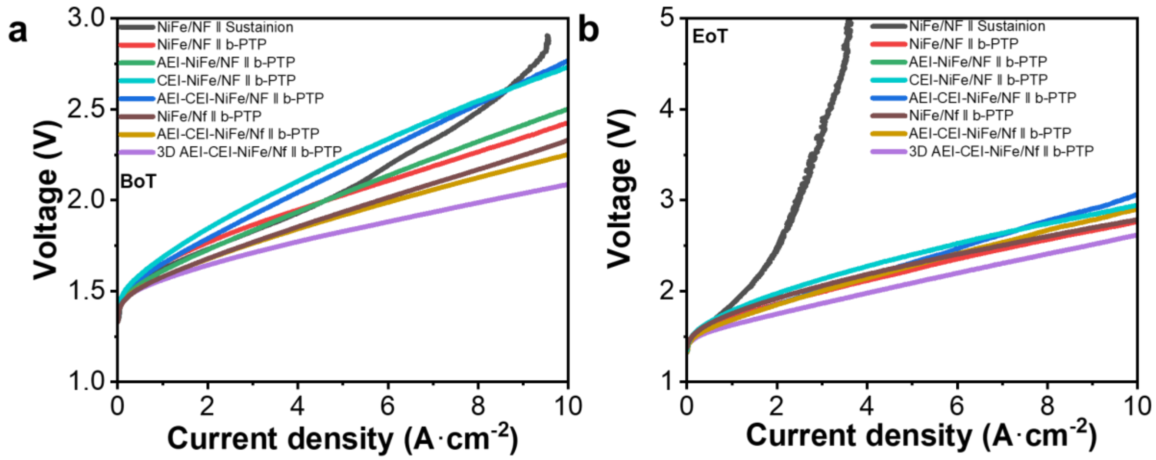

Supplementary Fig. 8 │ Transient LSVs at BoT and EoT. **a**, LSV curves at BoT. **b**, LSV curves at EoT.

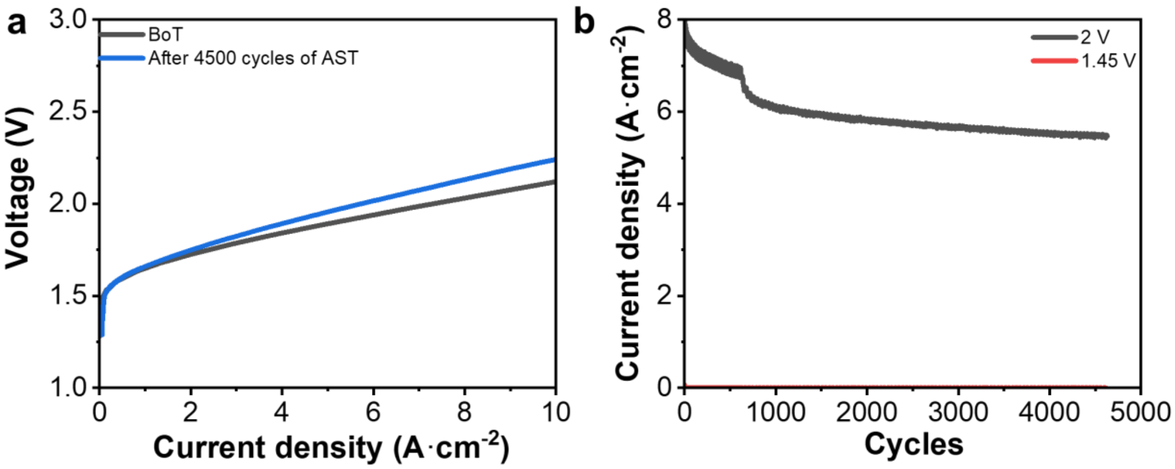

Supplementary Fig. 9 | 3D AEI-CEI-NiFe/Nf || b-PTP AEMWE under AST. **a**, LSV curves at BoT and after 4500 cycles of AST. **b**, dynamic current density response during AST.

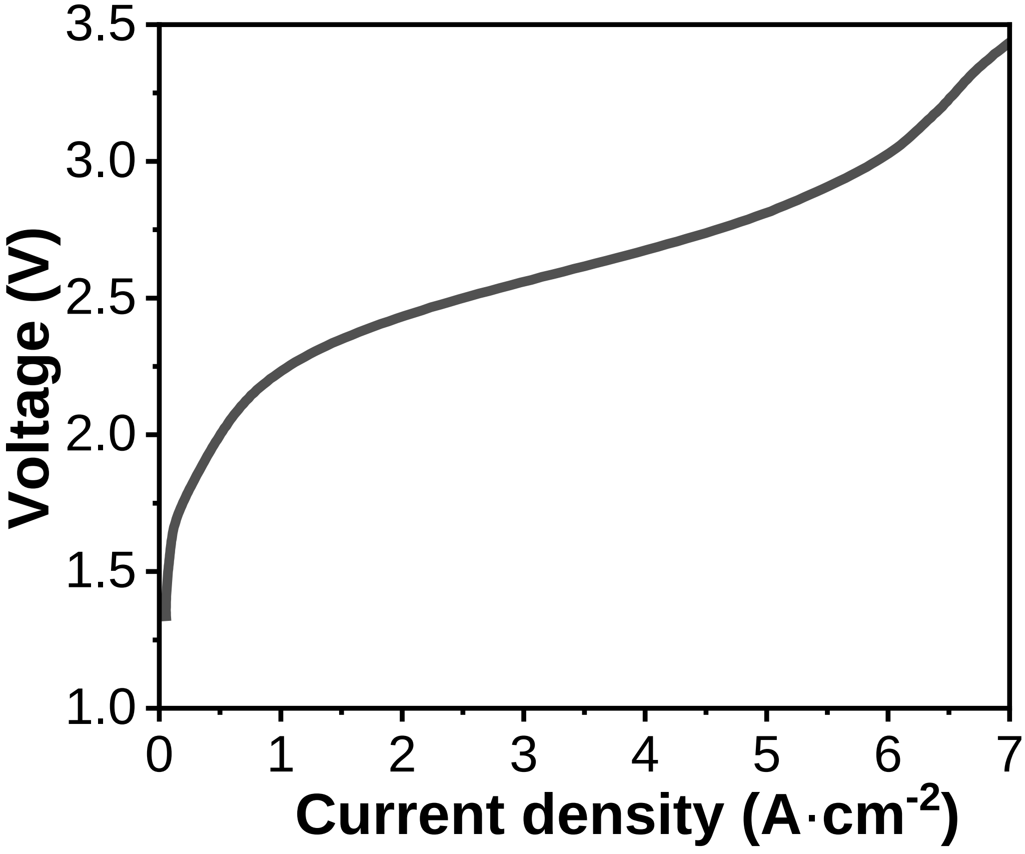

Supplementary Fig. 10 | 3D AEI-CEI-NiFe/Nf || b-PTP AEMWE under DI water operation: LSV curve at BoT.

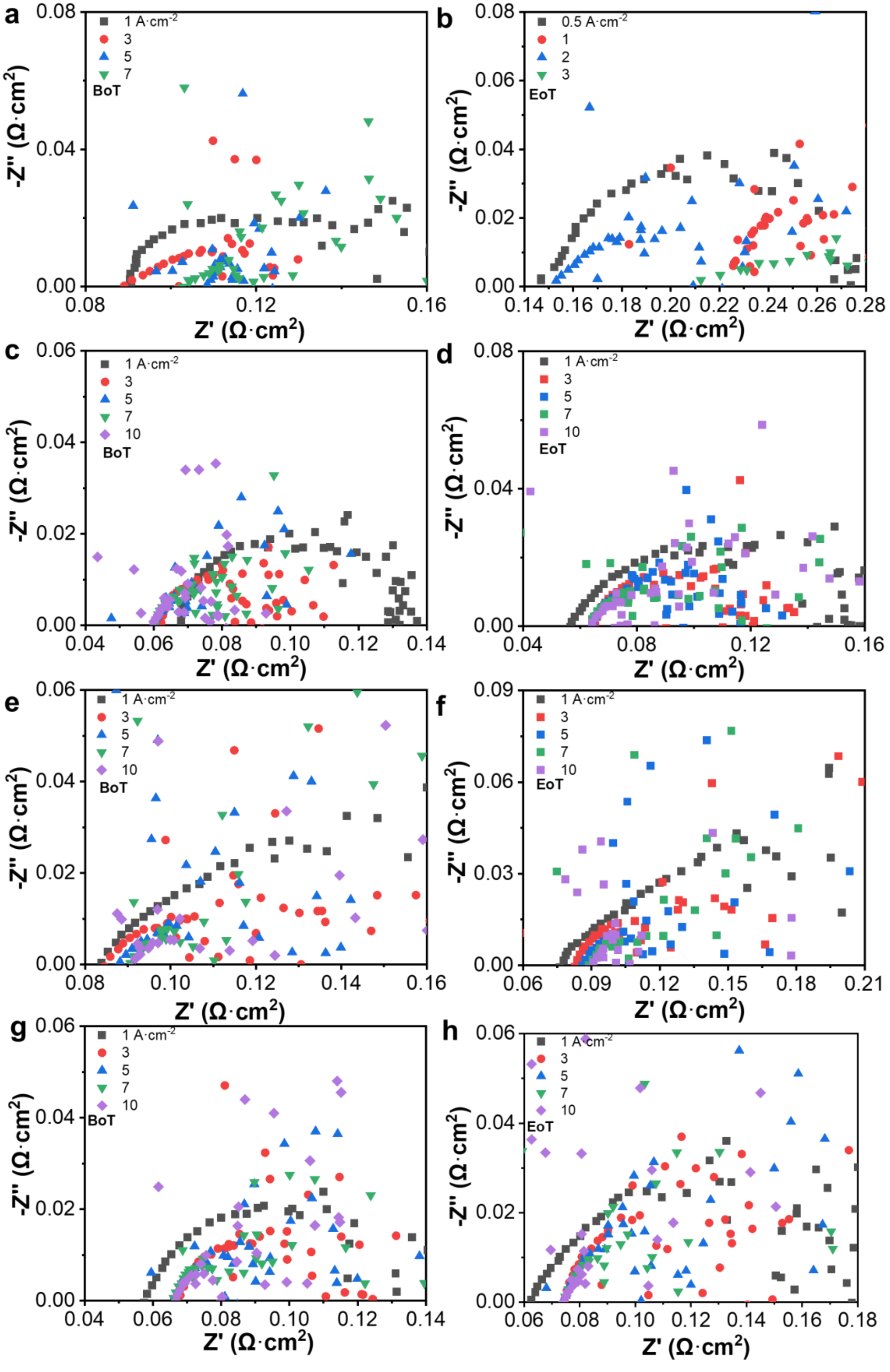
a
b
c
d
e
f
g
h
BoT
EoT
1 A·cm-2
0.5 A·cm-2
Z' (Ω·cm2)
-Z'' (Ω·cm2)

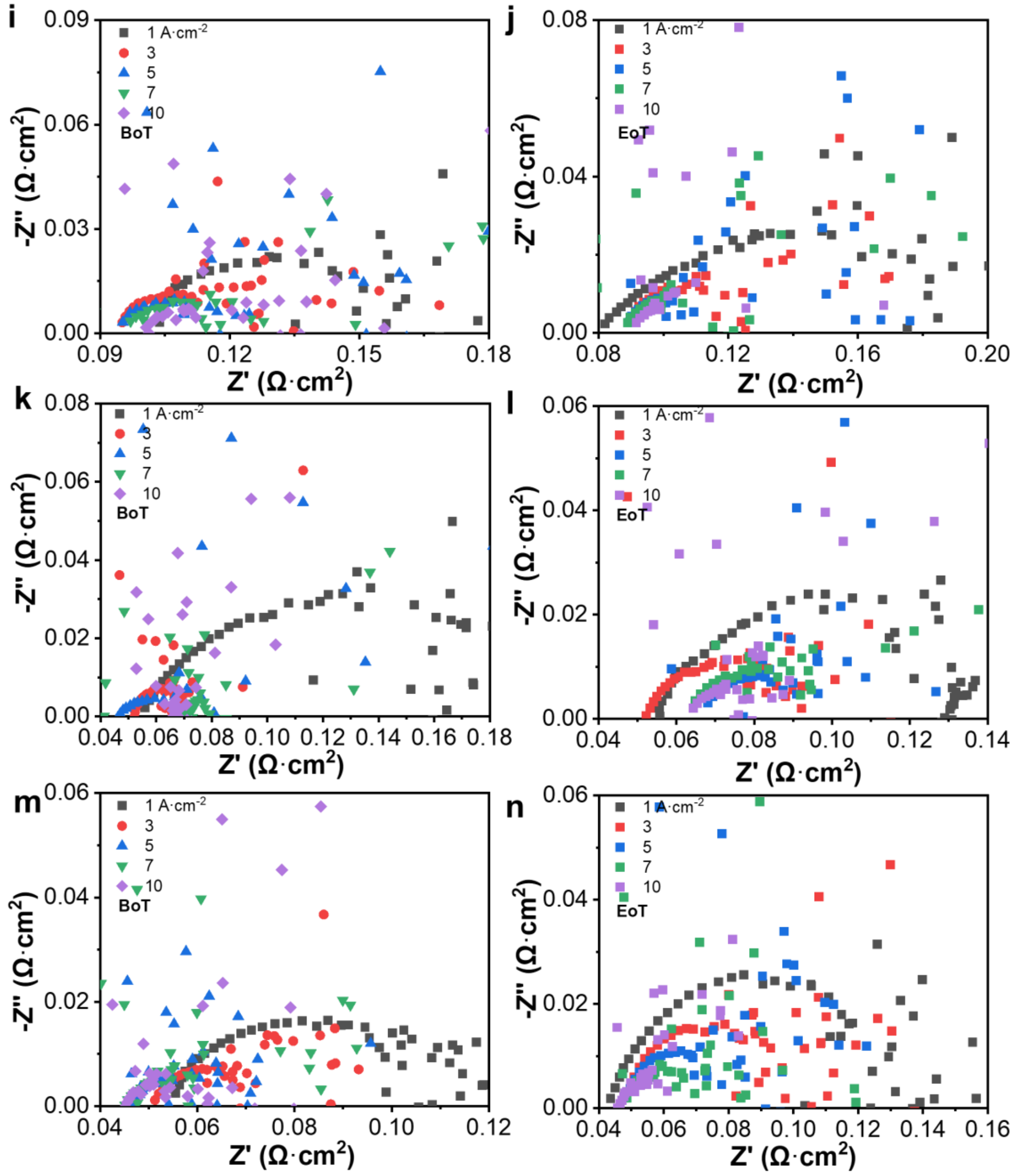

Supplementary Fig. 11│ EIS measurements upon changing current density (indictated by anodes || AEMs). a, NiFe/NF || Sustainion at BoT. **b**, NiFe/NF || Sustainion at EoT. **c**, NiFe/NF || b-PTP at BoT. **d**, NiFe/NF || b-PTP at EoT. **e**, AEI- NiFe/NF || b-PTP at BoT. **f**, AEI- NiFe/NF || b-PTP at EoT. **g**, CEI-NiFe/NF || b-PTP at BoT. **h**, CEI-NiFe/NF || b-PTP at EoT **i**, AEI-CEI-NiFe/NF || b-PTP at BoT. **j**, AEI-CEI-NiFe/NF || b-PTP at EoT **k**, AEI-CEI-NiFe/Nf || b-PTP at BoT. **l**, AEI-CEI-NiFe/Nf || b-PTP at EoT. **m**, 3D AEI-CEI-NiFe/Nf || b-PTP at BoT. **n**, 3D AEI-CEI-NiFe/Nf || b-PTP at EoT.

Supplementary Table. 1│ Ohmic resistances extracted from EIS upon changing current densities.

<table>
<tr><th>Parameters</th><th colspan="6">Ohmic resistance (Ω·cm^{-2})</th></tr>
<tr><td rowspan="4">NiFe/NF || Sustainion</td><td>J</td><td>1 A·cm^{-2}</td><td>3 A·cm^{-2}</td><td>5 A·cm^{-2}</td><td>7 A·cm^{-2}</td><td rowspan="4"></td></tr>
<tr><td>BoT</td><td>0.0900</td><td>0.0940</td><td>0.1004</td><td>0.1063</td></tr>
<tr><td>J</td><td>0.5 A·cm^{-2}</td><td>1 A·cm^{-2}</td><td>2 A·cm^{-2}</td><td>3 A·cm^{-2}</td></tr>
<tr><td>EoT</td><td>0.1505</td><td>0.2113</td><td>0.1591</td><td>0.2353</td></tr>
<tr><td></td><td>J</td><td>1 A·cm^{-2}</td><td>3 A·cm^{-2}</td><td>5 A·cm^{-2}</td><td>7 A·cm^{-2}</td><td><b>10 A·cm^{-2}</td></tr>
<tr><td rowspan="2">NiFe/NF || b-PTP</td><td>BoT</td><td>0.0671</td><td>0.0631</td><td>0.0614</td><td>0.0604</td><td>0.0608</td></tr>
<tr><td>EoT</td><td>0.0588</td><td>0.0661</td><td>0.0660</td><td>0.0657</td><td>0.0648</td></tr>
<tr><td rowspan="2">AEI-NiFe/NF || b-PTP</td><td>BoT</td><td>0.0985</td><td>0.0913</td><td>0.0947</td><td>0.0953</td><td>0.1023</td></tr>
<tr><td>EoT</td><td>0.0819</td><td>0.0889</td><td>0.0890</td><td>0.0895</td><td>0.0924</td></tr>
<tr><td rowspan="2">CEI-NiFe/NF || b-PTP</td><td>BoT</td><td>0.0580</td><td>0.0680</td><td>0.0663</td><td>0.0661</td><td>0.0671</td></tr>
<tr><td>EoT</td><td>0.0621</td><td>0.0740</td><td>0.0732</td><td>0.0736</td><td>0.0740</td></tr>
<tr><td rowspan="2">AEI-CEI-NiFe/NF || b-PTP</td><td>BoT</td><td>0.0839</td><td>0.0881</td><td>0.0905</td><td>0.0926</td><td>0.0927</td></tr>
<tr><td>EoT</td><td>0.0787</td><td>0.0845</td><td>0.0908</td><td>0.0938</td><td>0.0940</td></tr>
<tr><td rowspan="2">AEI-CEI-NiFe/Nf || b-PTP</td><td>BoT</td><td>0.0569</td><td>0.0532</td><td>0.0510</td><td>0.0720</td><td>0.0668</td></tr>
<tr><td>EoT</td><td>0.0560</td><td>0.0531</td><td>0.0700</td><td>0.0640</td><td>0.0617</td></tr>
<tr><td rowspan="2">3D AEI-CEI-NiFe/Nf || b-PTP</td><td>BoT</td><td>0.0535</td><td>0.0522</td><td>0.0474</td><td>0.0453</td><td>0.0460</td></tr>
<tr><td>EoT</td><td>0.0447</td><td>0.0487</td><td>0.0487</td><td>0.0485</td><td>0.0472</td></tr>
</table>

Supplementary Table. 2 | Cell voltages in stability tests at 10 A·cm^{-2}

	Voltage (V)
NiFe/NF || b-PTP	2.76
AEI-NiFe/NF || b-PTP	2.64
CEI-NiFe/NF || b-PTP	2.99
AEI-CEI-NiFe/NF || b-PTP	2.67
AEI-CEI-NiFe/Nf || b-PTP	2.51
3D AEI-CEI-NiFe/Nf || b-PTP	2.30

Supplementary References

[1] L. J. Titheridge, A. T. Marshall, *International Journal of Hydrogen Energy* **2024**, *49*, 518–532.
[2] N. Du, C. Roy, R. Peach, M. Turnbull, S. Thiele, C. Bock, *Chem. Rev.* **2022**, *122*, 11830–11895.
[3] L. Bai, S. Lee, X. Hu, *Angewandte Chemie* **2021**, *133*, 3132–3140.
[4] Y. Zheng, A. Serban, H. Zhang, N. Chen, F. Song, X. Hu, *ACS Energy Lett.* **2023**, *8*, 5018–5024.
[5] A. Allushi, P. M. Bakvand, P. Jannasch, *Macromolecules* **2023**, *56*, 1165–1176.
[6] A. G. Wright, J. Fan, B. Britton, T. Weissbach, H.-F. Lee, E. A. Kitching, T. J. Peckham, S. Holdcroft, *Energy Environ. Sci.* **2016**, *9*, 2130–2142.
